# Synovium extra cellular matrices seeded with transduced mesenchymal stem cells stimulate chondrocyte maturation *in vitro* and cartilage healing in clinically-induced rat-knee lesions *in vivo*

**DOI:** 10.1371/journal.pone.0212664

**Published:** 2019-03-12

**Authors:** Nathalie A. Reisbig, Erin Pinnell, Logan Scheuerman, Hayam Hussein, Alicia L. Bertone

**Affiliations:** Comparative Orthopedics Research Laboratory, Department of Veterinary Clinical Sciences, College of Veterinary Medicine, The Ohio State University, Columbus, Ohio, United States of America; University of Umeå, SWEDEN

## Abstract

Osteoarthritis (OA) is a progressive disease associated with cartilage injury and its inherently limited repair capability. Synovium-based cellular constructs (sConstructs) are proposed as possible treatments. Equine sConstructs were produced from decellularized synovium-based extracellular matrix scaffolds (sECM) seeded with synovium-derived mesenchymal stem cells (sMSC), and engineered to express green fluorescent protein (GFP), or bone morphogenetic protein-2 (BMP-2). Survival, distribution, and chondrogenic potential of the sConstructs *in vitro* and *in vivo* were assessed. sConstructs in co-culture with chondrocytes increased chondrocyte proliferation, viability, and Col II production, greatest in BMP-2-sConstructs. Chondrocyte presence increased the production of hyaluronic acid (HA), proteoglycan (PG), and BMP-2 by the sConstructs in a positive feedback loop. sECM alone, or GFP- or BMP-2-sConstructs were implanted in synovium adjacent to clinically created full-thickness rat-knee cartilage lesions. At 5 weeks, the lesion area and implants were resected. Gross anatomy, adjacent articulate cartilage growth and subchondral bone repair were scored; and peripheral, central and cartilage lesion measurements taken. For all scores and measurements, sConstruct implants were significantly greater than controls, greatest with the BMP-2-sConstructs. Immunohistochemistry demonstrated migration of endogenous cells into the sECM, with greater cellularity in the constructs with intense positive GFP staining confirming engraftment of implanted sMSC and continued gene expression. In summary, exposing cartilage to sConstructs was chondrogenic *in vitro* and *in vivo*, and resulted in substantially increased growth *in vivo*. This effect was mediated, in part, by soluble ECM and cell factors and upregulation of anabolic growth proteins, such as BMP-2. This work is “proof of concept” that sConstructs surgically implanted adjacent to cartilage damage can significantly improve cartilage and subchondral bone repair, and potentially prevent the progression of OA.

## Introduction

Osteoarthritis (OA) has the highest disability rate and health cost of any single disease in the United States [1]. It is an irreversible degenerative joint disease characterized by articular cartilage loss and synovial inflammation [2]. The most widely used surgical treatment, micro-fracture, results in weak fibro-cartilage repair tissue even after long convalescence [3–5]. New surgical treatments, such as Autologous Chondrocyte Implantation (ACI) [6] and Osteochondral Autograft Transfer System (OATS), are used to repair cartilage by directly grafting the injured cartilage site [7]. Although both methods have shown good outcomes, cell yield in the grafts is low and the new tissue is still inferior in quality. A long period of rehabilitation is necessary to avoid overload of the avascular grafted cartilage and is a significant limitation. The grafted injured site does not receive a continued, renewable source of bioactive anabolic mediators. We have suggested a different strategy to overcome this weakness [8]: Use of a microenvironment paracrine-like feedback loop via a bioactive transplant in the synovium nearby a cartilage injury. Such a transplant, made from synovium components (sConstructs) has not, to our knowledge, been reported in the literature.

Synovium is a rich source of both pericyte and tissue-specific mesenchymal stem cells (MSCs) [9–11]. We selected synovial MSCs (sMSCs) for our study to both retain the potential to differentiate to other tissue types (cartilage) for migrating cells, and rapid phenotypic differentiation and production of supportive joint-specific biomediators, such as hyaluronic acid (HA). Other studies have shown that MSCs in joints due produce biomediators [6,12,13]. Treatments using autologous MSC solutions have tried to overcome the lack of growth nutrients around the damaged area [6]. MSCs injected into the joint improved cartilage repair, but MSCs were not found in the lesions [13]. These data support that MSCs produce biomediators that enhance the growth environment.

To enhance the efficacy of MSCs, cells have been placed in the damaged area using a MSC-seeded scaffold, gel, or aggregate implant [12,14–16]. Cartilage, a natural choice for a scaffold, has been a suboptimal scaffold for healing joint injury [15]. Current thought is that a synthetic scaffold using biological-derived MSC or chondrocyte constituents may grow and mature along with the regenerating cartilage. The bio-construct could provide the appropriate growth environment over the whole healing period. These approaches still placed the bio-scaffolds in, or on, cartilage sites.

Many investigations have focused on developing a synthetic bio-constructs that mimic a natural scaffold that is still placed in, or on, cartilage [17]. We have taken the unique approach of using synovium tissue to make a natural scaffold [18] and potentially heal nearby cartilage. We selected synovium-origin tissue for our ECM scaffolds. The biological nature of sECMs should retain the porosity of the native tissue, the collagen components, cell surface receptors such as integrins, serve as a reservoir for growth factors, and provide a substrate for cell attachment and migration [19]. Additionally, *in vitro*, ECMs have been shown to encourage the formation of tissue specific phenotypes [20,21]. We have reported our decellularization procedures to produce synovium-derived extra-cellular matrix (sECM) [8] and our sMSC seeding procedures to show that sMSC will retain viability, distribute, proliferate, differentiate and produce soluble biomediators, including inserted transgenes, such as the chondrogenic bone morphogenetic protein two (BMP-2) [18]. BMP-2 was chosen because of its strong anabolic properties and our laboratory’s access and more than a decade of work with these reagents [22]. Indeed, we confirmed *in vitro* that sConstructs can produce greater concentrations of the growth factor BMP-2 from transduced sMSCs seeded into sECMs (BMP-2-sConstructs) compared to untransduced-sConstructs [18]. Use of sMSCs transduced with green-fluorescent protein (GFP) was included in these studies for cell tracking, confirmation of successful transduction, *in vitro*. [17,18]

A few reports, mostly *in vitro*, have demonstrated that MSCs can be chondrogenic [23]. Synovium-origin MSCs have been shown *in vitro* to have a superior effect on chondrogenesis over all other MSCs [11,24]. Here, in this study, we have created an *in vitro* experimental design to show “proof of concept” that our sConstructs, made from synovium, could influence chondrocytes when not in direct contact by communicating via soluble factors in fluid (media). This co-culture model mimics the situation in joints with the synovium and cartilage communicating only via synovial fluid since articular cartilage is avascular. This *in vitro* system also can determine the presence of a feedback loop in which stimulated chondrocytes could further stimulate the sConstruct. Chondrocyte morphology, cell viability, and intracellular Collagen Type II (Col II) were analyzed. The chondrocyte impact on sMSCs in sConstructs was followed by measuring the sMSC’s growth in cell counts, viability, and BMP-2 production. In addition, sMSC maturation is quantified by determining the concentration of soluble biofactors, hyaluronan (HA) and proteoglycan (PG) excreted into the medium. Once demonstrated, we proceeded with *in vivo* studies.

Using our experience with the nude rodent model to study knee cartilage healing [25,26] we determined the healing potential of the sConstructs. The time point of healing, 5 weeks, was selected such that we could detect either a delay or acceleration of repair among our comparison groups. These rat knee lesions generally heal around 8 weeks. Longer duration studies run the risk of missing the window such that the lesions could all be healed. For our *in vivo* studies, sConstructs were implanted in the sub-patellar synovium directly adjacent to clinically-produced, athymic-nude-rat-knee lesions on the femur. After a 5-week healing period, knees were resected and lesion morphology, fibrocartilage repair tissue, subchondral bone repair, and cartilage growth are examined and quantitated. Host lesion effects on sConstructs was determined by *en bloc* resection of the synovium implant site, evaluating the morphology, and tracking sMSC cell integration.

Our overall hypothesis was that sConstructs would enhance both chondrogenesis *in vitro* and cartilage repair *in vivo*. Additionally, BMP-2-sConstucts would be superior to the bioactivity of other sConstructs. If this hypothesis is correct, then this study offers a “proof of concept” that sConstructs and their derivative transduced-sConstructs are an alternative therapy for OA.

## Materials and methods

### General experimental methods

The following general methods were used through all the experiments. Cells were cultured under sterile conditions, at 37°C in Dulbecco’s Modified Eagle’s Medium (DMEM), supplemented with penicillin,100 U/mL, streptomycin, 100 μg/mL, and amphotericin 250 ng/mL (supplemented DMEM, Gibco, Gaithersburg, MD) and 10% fetal bovine serum (FBS, Sigma Aldrich, Steinheim, Germany), in T75 flasks with shaking. This supplemented DMEM was used in all cell cultures unless specified otherwise. For co-culture studies, Transwell 12-well culture plates with snap-in 6 mm diameter inserts designed for the plates were used (Transwell Culture Insert, Corning Costar, Sigma Aldrich, Steinheim, Germany). Each lower well held approximately 0.6 mL of solution, and the insert held approximately 0.1 ml. Cells were counted on a hemocytometer or by flow cytometry done on an Accuri Cytometer (Ann Arbor, MI). Enzyme-linked immunosorbent assay (ELISA) kits followed the instructions of the vendors.

### In vitro study design

Cell and tissue preparation began 30 days before the start of the *in vitro* study with the fresh harvest of synovium and cartilage tissues for preparation of the sECM, sMSC, and isolated chondrocytes (Table 1) [8,18]. Once prepared, all cells and scaffolds were frozen and thawed prior to the start of the experiments that started on Day 0 on the time line detailed in Table 1. All experiments were performed comparing our groups, described later, both with and without the chondrocyte monolayer culture in the bottom well. The *in vitro* study harvested media and specimens on Day 3,7, and 14 to study early influence among the biologics and mimic the time anticipated for clinical application (within 2 weeks) for our *in vivo* portion of the study. For each study with and without chondrocytes, nine replicates were performed (three horses, 3 samples each in triplicate) with allogeneic assignment of sMSC and sECM.

**Table 1:** Preparation prior to *in vitro* co-culture experiment start Day 0.

Day Cells/Scaffolds	< -30 days Biologic Preparation	-8 days sMSC transduction	-6 days	-5 days sConstruct preparation	-3 days Chondrocyte preparation	Day 0 Experiment start
sECMs	sECM decellularized from fresh synovium, cut into 6 mm ⊘ discs and frozen (-80°C).			sECM discs thawed at room temperature for 3 hrs and placed in the 6 mm ⊘ insert.		sECM inserts followed alone and placed into plate wells containing chondrocytes
sMSCs	sMSCs prepared from fresh synovium and passaged.	sMSC transduced with Ad-BMP2.	ELISA for BMP2 in the Media from sMSC-BMP2. confirmed transduction and BMP2 greater than sMSC.	10^6^ viable cells in 10% FBS were placed on sECM in insert.		
Chondrocytes	Chondrocytes isolated from digestion of fresh cartilage and frozen at -80°C.				Chondrocytes thawed, 10^3^ cells in 10% FBS were placed into each well of the 12 well plate for the experiments assigned to co-culture. Medium was changed daily.	Chondrocytes followed alone and in the plate wells of co-cultures
sConstruct				Inserts were placed into 12 well plates containing 40% FBS. Medium replaced daily, 10% FBS in insert, 40% FBS in well.		sConstruct inserts were placed into plate wells containing chondrocytes.

#### Chondrocyte, sMSC and sECM preparation

Equine villous synovium from the medial femoral patellar joint and articular cartilage from the femoral condyles were harvested [8] from knee joints of healthy adults (2–7 years old), euthanized for reasons unrelated to this study. Briefly, the joints were aseptically prepared with 2 5min scrubs using 4% chlorhexidine and sterile H_2_O. Sterile gloves and instruments were used to enter and harvest tissue. The joints were macroscopically inspected for any abnormalities and excluded if any were noted. Chondrocytes and sMSCs were purified from the cartilage and synovium aseptically [18,27], divided into small pieces and digested in 0.02% collagenase type II solution (Sigma Aldrich, Steinheim, Germany) at 37°C for 5 hours; cells were filtered (70- μm cell strainer), and cultured. Cell monolayers were grown to a > 90% confluence and passaged 3 to 6 times prior to use. This ensured a uniform population of sMSCs in which >95% of the cells express a multipotent MSC phenotype with minimal presence of lymphocytes, natural killer cells and macrophages [28,29]. Caplin et.al. [10] have shown that prepared MSCs can consist of tissue phenotype sMSC and perivascular cells, pericytes, pMSCs. No attempt here has been made to quantify nor classify the pMSCs from the tissue sMSCs. sECM was decellularized from harvested synovium by immediately dividing the tissue into 1x1 cm sheets using a dissecting microscope, then incubating them at 37°C in 0.1% peracetic acid (PAA) and 4% ethanol for 6 hours with mechanical agitation twice [8]. Samples of sECM were prepared using a 6 mm diameter biopsy punch, frozen wet at –80°C, surrounded by dry ice in ethanol. All the components were used within 30 days of preparation.

#### sMSC transduction

Replication-deficient, E1-A-deleted adenoviral (Ad) vectors encoding for a 1547 base-pair open reading frame segment of human bone morphogenic protein-2 (Ad-BMP-2) under the control of the cytomegalovirus promoter were generated [30], titered by plaque assay, and stored at -80°C. sMSCs to be transduced were incubated at a multiplicity of infection (MOI) of 100 [27] with Ad-BMP-2. On day -5 prior to the experiment, the untranduced and transduced cells (BMP-2-sMSC) were evaluated for BMP-2 expression by ELISA, the percent of viable cells and then suspended to a concentration of 1.0 × 10^6^ cells/mL.

#### sConstructs preparation

Approximately 50 sECM samples from each of three horses were thawed to have three different biologic samples run in triplicate distributed for each group: controls (sECM alone) and two sConstruct types; sConstructs with 1) untransduced sMSCs (untransduced-sConstructs) and 2) BMP-2 transduced sMSC (BMP-2-sConstructs), seeded with 1 x10^6^ untransduced or transduced-sMSCs, respectively. A 30% FBS, gradient from insert to well was made by having 10% FBS in the insert and 40% FBS in the well to attract the sMSC to seed deep within the sECM [31]. The gradient was maintained by replacing the medium in the inserts and wells every 24 hrs. The seeding was allogeneic; sECMs from one animal were never seeded with the same animal´s sMSCs.

#### Chondrocyte preparation and experiment start

Single passaged Chondrocytes (1 x 10^3^ chondrocytes/well) were cultured in 10% FBS for 3 days in 12 well culture plates prior to the experiment. The experiment was initiated (day 0) when Transwell inserts containing sECM or sConstructs were placed into the wells with the cultured chondrocytes. Media, approximately 0.7 ml, from the wells and Transwell inserts was collected daily (stored at -20°C) and replaced with fresh media, 10% FBS supplemented DMEM.

### In vitro sConstruct-chondrocyte co-culture assays

Table 2 is a summary of the assays performed on the chondrocytes-sConstructs co-cultures on days 3, 7, and 14.

**Table 2:** Assays performed on 3,7, and 14 days.

Characteristic	Assay type	Sample preparation
sConstructs*		
sMSC cell count, viability & maturation	Flow cytometry Microscopy (hemocytometer)	sConstructs removed from inserts and digested with collagenase. Labelled 7AAD and CD90 for flow and trypan blue vital stain for microscopic count.
Medium**		
Soluble HA, PG, & BMP-2 conc.	ELISA	Every 24 hrs, medium from inserts and wells was collected and pooled for three time points; 0–3, 4–7, 8-14-day samples.
Chondrocytes***		
Cell count & viability	Flow cytometry Microscopy (hemocytometer)	Removed medium. Cells in wells were lifted with trypsin. Labelled 7-AAD for flow and trypan blue vital exclusion stain for microscopic count.
Coll II concentration	ELISA	Removed medium. Cells in wells were lysed and effluent assayed.
Cell morphology	Microphotometry	Removed medium, fixed cells in well, and toluidine blue stained. Cell monolayers were photographed under controlled conditions.

* Number of samples per characteristic = 3 horses x 3 samples/horse x 3 replicates x 3 co-culture groups (sECMs, untransduced-sConstructs, BMP-2-sConstructs) x 3 time periods = 3 x 3 x 3 x 3 x 3 = 243 samples.

** Number of samples per characteristic (HA, PG, BMP-2) = same as sConstructs except 4 co-culture groups (additionally media from chondrocytes alone) = 3 x 3 x 3 x 4 x 3 = 324 samples

***Number of samples per characteristic = 3 horses x 3 samples/horse x 3 replicates x 4 co-culture groups (chondrocytes alone, sECMs, untransduced-sConstructs, BMP-2-sConstructs) x 3 time periods = 3 x 3 x 3 x 4 x 3 = 324 samples.

#### Chondrocyte and sMSC cell counts, viability, and maturation

Cells on culture plates were trypsinized. sECMs and sConstructs from inserts were digested with collagenase as described above for synovium and cartilage. Cells were counted with a hemocytometer and flow cytometry. Cell viability was determined by detecting the fraction of cells stained with 7-aminoactinomycin D, (7AAD, BD Biosciences, San Diego, CA). Cells were incubated with 7AAD for 2 hours, washed twice with PBS solution, and analyzed by flow cytometry. SMSCs maturation was determined by % cluster differentiation 90 (CD90). After cells were counted, 5 X 10^6^ cells were combined with 0.2 μL of anti-human CD90 primary monoclonal antibody (Clone 5E10 directly conjugated to phycoerythrin, BD Bioscience, San Diego, USA), incubated at 37°C for 2 hours, washed, resuspended in 200 μL of PBS, and analyzed using flow cytometry.

#### HA, PG, BMP-2 and Coll II determinations

ELISA kits were used as per manufacturer’s recommendations to determine the concentrations of HA (Hyaluronan Quantikine, R&D Systems, Minneapolis, MN), PG (Proteoglycan assay kit, Rheumera, Redmond, WA), BMP-2 (BMP2 Quantikine, R&D Systems, Minneapolis, MN), and Col II (MyBioSource, Inc., San Diego, CA). For HA, PG and BMP-2 co-culture medium from the insert and well was collected daily, frozen to -20°C, pooled for days 1–3, 4–7, and 8–14 then analyzed. For Col II concentrations, chondrocytes in the wells were lysed and effluents analyzed.

#### Morphology of sConstructs and chondrocytes from co-cultures

sECM and sConstructs from inserts were formalin fixed, thinly sectioned (8 μm), and stained with hematoxylin and eosin (H&E) for subjective microscopic evaluation for architecture, cellularity and density of the eosin staining. For the chondrocyte morphology, the Transwell inserts were removed from the culture plates and the chondrocyte monolayers in the wells were fixed, stained with toluidine blue and microphotographed under standard conditions. The microphotographs were scored from 0 to 4 with 0.5-point increments for phenotype (Table 3) by a trained investigator blinded to the treatment group [32]. One score was determined from a consensus score from multiple areas in the well.

**Table 3:** Scoring criteria for chondrocyte morphology, lesion gross anatomy, adjacent articulate cartilage growth and subchondral bone repair.

Score	Chondrocyte Morphology	Gross Anatomy	Adjacent Articulate Cartilage Growth	Subchondral Bone Repair
4 & 3.5	> 75% of cells with chondral morphology typical of a hypertrophied cell or chondroblast (cellular enlargement and rounded shape with greater cytoplasm-to-nucleus ratio) < 25% of cells with early chondral morphology (elongated, tapered, spindle- or stellate- shaped cells, with relatively low cytoplasm-to-nucleus ratio; confluence often leads to mounding of cells)	Intact cartilage surface	No abnormalities Normal morphology Normal cellularity Intense toluidine blue stain Normal thickness	No damage Reestablished tidemark Reestablished subchondral bone plate Calcified zone of cartilage reformed No fragmentation No bone marrow changes
3 & 2.5	50 to 75% of cells with chondral morphology typical of a hypertrophied cell or chondroblast 25 to 50% of cells with early chondral morphology	Surface roughening No presence of cartilage lesions or osteophyte formation	Normal cellularity >75% cartilage thickness Intense toluidine blue stain Lacunae doublets present No chondrone formation	No marrow changes or, if present, minimal and focal Increased thickening of subchondral bone subjacent to the area of greatest articular cartilage lesion severity.
2 & 1.5	25 to 50% of cells with chondral morphology typical of a hypertrophied cell or chondroblast 50 to 75% of cells with early chondral morphology	Deeper defects (fibrillation, fissures) not involving the subchondral bone Mild osteophyte formation	Moderate cellularity 50–75% cartilage thickness Moderate Intensity of toluidine blue stain No lacunae doublets No chondrone formation	Minimal to mild focal fragmentation of calcified cartilage of tidemark, Mesenchymal change in marrow (fibroblastic cells) involving about 1/4 of subchondral region under lesion Increased thickening of subchondral bone subjacent to the area of greatest articular cartilage lesion severity
1 & 0.5	25% to 50% of cells with early chondral morphology < 25% of cells with chondral morphology typical of a hypertrophied cell or chondroblast	Erosions down to subchondral bone (less than 5 mm diameter) Presence of multiple osteophytes	Low cellularity 25–50% cartilage thickness Low intensity of toluidine blue stain No lacunae doublets Chondrone formation	Mild to marked fragmentation (multiple larger areas) of calcified cartilage/subchondral bone loss Mesenchymal change in marrow up to 3/4 of total area, Areas of marrow chondrogenesis may be evident No major collapse of articular cartilage into epiphyseal bone (definite depression in surface).
0	No chondral morphology < 25% of cells with early chondral morphology	Large erosions down to subchondral bone Osteophyte formation	No cellularity Low toluidine blue stain No lacunae doublets Chondrone formation <25% cartilage thickness	Marked to severe fragmentation of calcified cartilage Mesenchymal change in marrow > 3/4 of area Articular cartilage has collapsed into the epiphysis to a depth of 250 mm or less from tidemark

### In vivo rat lesions

#### Rat knee lesions with sConstruct implants

Twelve female athymic nude rats (10–12 weeks of age; 24 knees, Charles River Laboraories, Wilmington, MA) were used with four groups (n = 6 knees each), suture alone (control), sECM alone, GFP- or BMP-2-sConstructs (created as described above), following an approved Institutional Animal Care and Use protocol. Implants were randomized to different knees and rats. After the rats had 1 week of acclimation, anesthesia was induced with 5% isoflurane (GlaxoSmithKline, Research Triangle Park, NC), then maintained with 1% isoflurane delivered via nose cone. The surgical site was aseptically prepared and draped. Each knee had bilateral surgery; a full-thickness articular cartilage lesion was created in the intertrochlear groove of the femur until subchondral bone was penetrated, representing a marrow-stimulating technique. Specifically, a medial parapatellar approach was performed and the trochlear groove exposed by sub-luxating the patella. In the trochlear groove, a cartilage lesion, 2.5 mm diameter by ≈ 1.5 mm depth, was created using a motorized burr (Dremel, Mount Prospect, IL). In the control group (6 knees), suture was placed in the sub-patellar synovium directly adjacent to the trochlear groove. Constructs, prepared as described above, were removed from the Transwell inserts and cut to a 3 mm diameter with a biopsy punch. The assigned sConstruct implant was sutured (6–0 non-absorbable) to the synovium, similar to the control suture, directly adjacent to the lesion. The arthrotomy was closed with absorbable suture (Polydioxanone, Ethicon, Bridgewater, NJ). Buprenorphine (GlaxoSmithKline, Research Triangle Park, NC) 0.05 mg/kg was administered intramuscularly for pain. Prophylactic Augmentin (GlaxoSmithKline, Research Triangle Park, NC) 0.35 mg/mL was given 1 day preoperatively and 2 weeks postoperatively in the animals’ drinking water. The rats could roam freely in their cage. Rats were observed daily for incision swelling or drainage, chewing, ambulation, appetite and changes in behavior.

#### Gross anatomy, articular cartilage growth, subchondral bone repair scores and lesion filling measurements

**Fig 1:**
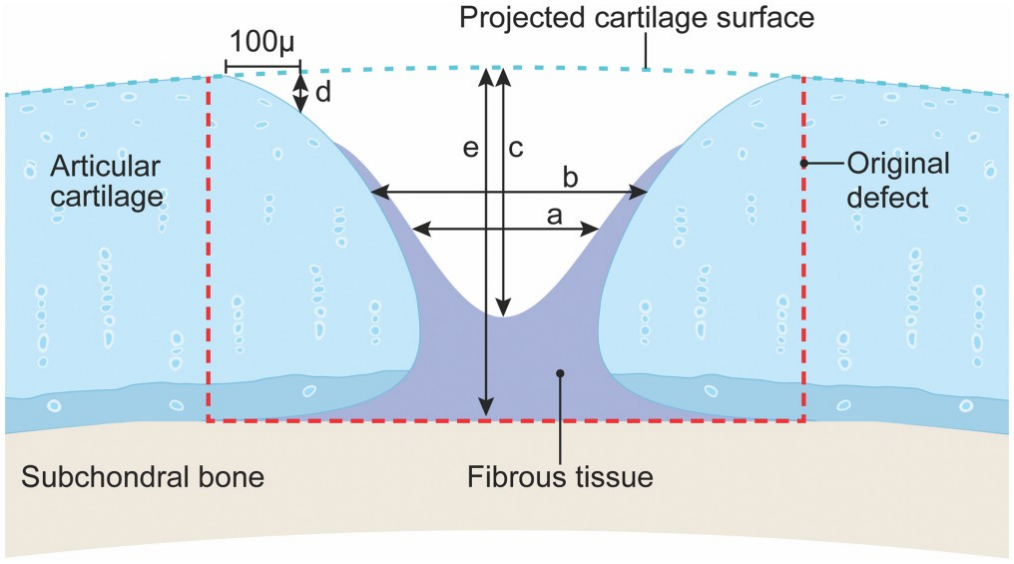
Histomorphometric measurements of lesion filling. Five measurements were taken for lesion filling a) width of opening halfway between the lesion bottom and the projected original surface b) width, at the same depth as measurement a, of opening of physiological cartilage, c) depth of the opening at the center, d) depth at 100μm from edge of original lesion, and e) depth of original lesion, from the projected cartilage surface to the estimated original tidemark. Red dashed line represents the size of the original lesion (2.5 mm wide x ~ 1.5 mm deep).

After 5 weeks, rats were euthanized with CO2. Rats had no sign of infection or lameness at any time. The articular surface of the knees was examined grossly and photographed for gross anatomy scoring. For articular cartilage repair and subchondral bone damage scores and all measurements, the distal femur was resected en bloc, fixed in 10% neutral-buffered formalin (NBF, Sigma Aldrich, Steinheim, Germany) for 48 hours, decalcified (Decalcifier-S, U.S. Biotex, Webbville, KY) for 12 hours, paraffin-embedded, and serially sectioned, 30 μm apart, in the sagittal plane, and stained with toluidine blue. Scoring was performed by two independent investigators (NR and ALB) blinded to implant group. Scoring criteria was a modification of an OA scheme described by Gerwin et al. [33] (Table 3). Scores ranged from 0 to 4, where 4 was growth like original tissue and 0, maximum damage. Five measurements were taken for lesion filling (Fig 1); a) width of opening halfway between the lesion bottom and the projected original surface, b) width, at the same depth as measurement a, of opening of physiological cartilage, c) depth of the opening at the center, d) depth at 100μm from edge of original lesion, and e) depth of original lesion, from the projected cartilage surface to the estimated original tidemark. All measurements were obtained using imaging software (Image J, NIH) and the reported values of all scores and measurements was the mean of three consecutive sections [34].

#### Lesion-sConstruct morphology

Implants were identified in the synovium by the suture and cut out en bloc, then carefully dissected free, fixed in neutral buffered formalin, paraffin-embedded, sectioned (7μm), stained with H&E. GFP Immunohistochemistry was done as previously described [35]. In brief, formalin fixed synovial biopsy specimens were sectioned (4 μm) and stained with polyclonal rabbit anti-human GFP antibody at a dilution of 1:100 and counterstained with biotinylated goat anti-rabbit anti- body (Vector Laboratories, Burlingame, CA) at a dilution of 1:200. then analyzed subjectively for cellular content, integration and tissue density.

### Statistics

There was no significant difference among horses, among samples or among technical replicates (P>0.05).

Numeric continuous outcomes (CD90 expression (%); cell counts, % viability, HA, PG and BMP-2 concentrations) were analyzed by means of the Shapiro-Wilk method to confirm normality of variance followed by a 2-way ANOVA (group and time) and post-test multiple comparisons using Proc Mixed statistical models (SAS Institute Inc, Cary, NC).

The ordinal categorical outcomes *in vitro* chondrocyte morphology, and *in vivo* gross anatomy, smooth fibrocartilage repair, and subchondral bone repair were examined using Genmod statistical models. Repeated variables were considered nested with animal as a random factor.

The *in vivo* cartilage histomorphometric data (lesion width, depth, and percentages) were examined for normality of variance (Shapiro-Wilks test) and the data were examined using linear regression within multi-level mixed-effects models with treatment groups as a fixed factor, controlling for rat, leg and slide replicate within the model.

High intra-observer (Pearson r = 0.92–0.99) and inter-observer (intra-class correlation, 0.94–0.99) have been reported demonstrating reliability of our elementary and complex histologic scoring systems for articular cartilage repair [36,37]. The intra-observer correlation was high (Pearson r = 0.91–0.99). Power calculations were made using means, standard deviations, sample sizes and 5% alpha-error level of all outcomes that differed significantly by treatment groups.

Significance level was set at *p* < 0.05 for all analyses. Data were expressed as mean ± SEM.

## Results

For all the studies, sMSCs prior to seeding had a cell count of 1.0 x10^6^ cells per scaffold, a cell viability as measured by 7AAD > 90%, a CD90 expression > 70%, and, for sMSC-BMP2 a soluble BMP-2 mean of 1.04 x 10^−5^ ng/cell. For the in vivo studies, the transduced GFP-sConstucts were used as an additional control for transduced BMP-2-sConstructs. The GFP- and BMP-2-MSCs had transduction efficiencies > 58% at 100 MOI. In all the samples, there was no significant difference among the different horses.

### In vitro studies

#### sConstructs impact on chondrocytes

**Fig 2:**
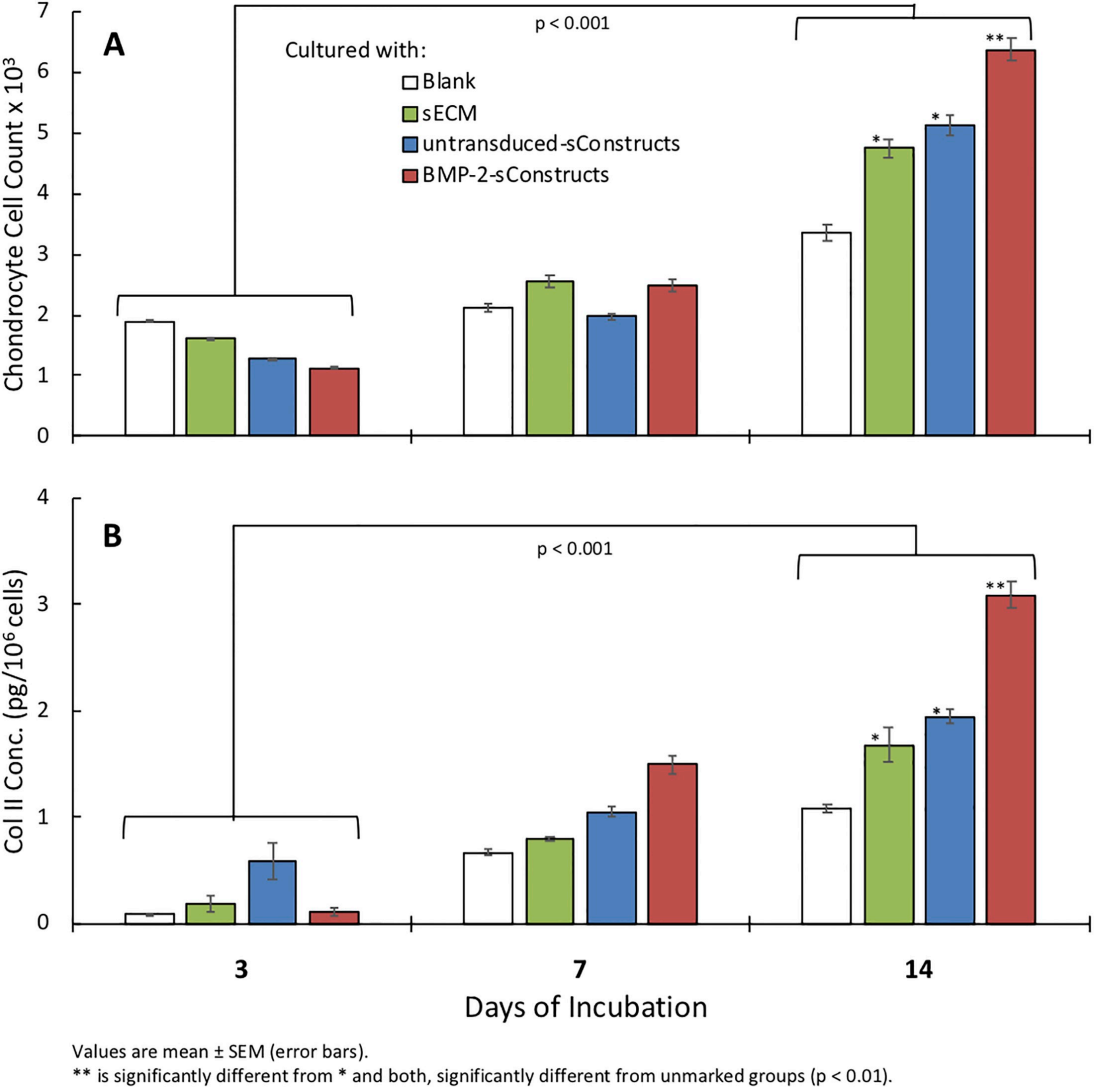
Co-culture chondrocyte cell counts and intracellular Col II production. Cell counts (A) and Col II concentration (B) from chondrocytes cultured alone (white), with sECM (green), untransduced-sConstructs (blue) or BMP-2-sConstructs (red) on day 3, 7, and 14.

Co-cultured chondrocyte cell counts were affected by sConstructs (Fig 2A). At day 3, chondrocyte cell counts were inhibited by BMP-2-sConstructs > untransduced-sConstructs > sECM. This was reversed by day 14 when chondrocytes co-cultured with BMP-2-sConstructs had a 1.9-fold (p < 0.001) increase in counts over chondrocytes alone. sECMs and untransduced-sConstructs enhanced chondrocyte counts 1.4-fold and 1.5-fold fold (p < 0.001), respectively, with no significant difference between them. Co-cultured chondrocyte cell viability, for all samples remained above 83% and was not significantly affected by co-culturing with sConstructs. Chondrocyte intracellular Col II concentration mirrored the cell count measurements (Fig 2B). After 14 days, Col II increased 2.9- (p <0.001), 1.8- (p, 0.001), and 1.6-fold (p, 0.001) in chondrocyte co-cultures with BMP-2-sConstructs, untransduced-sConstructs, and sECMs, respectively.

**Fig 3:**
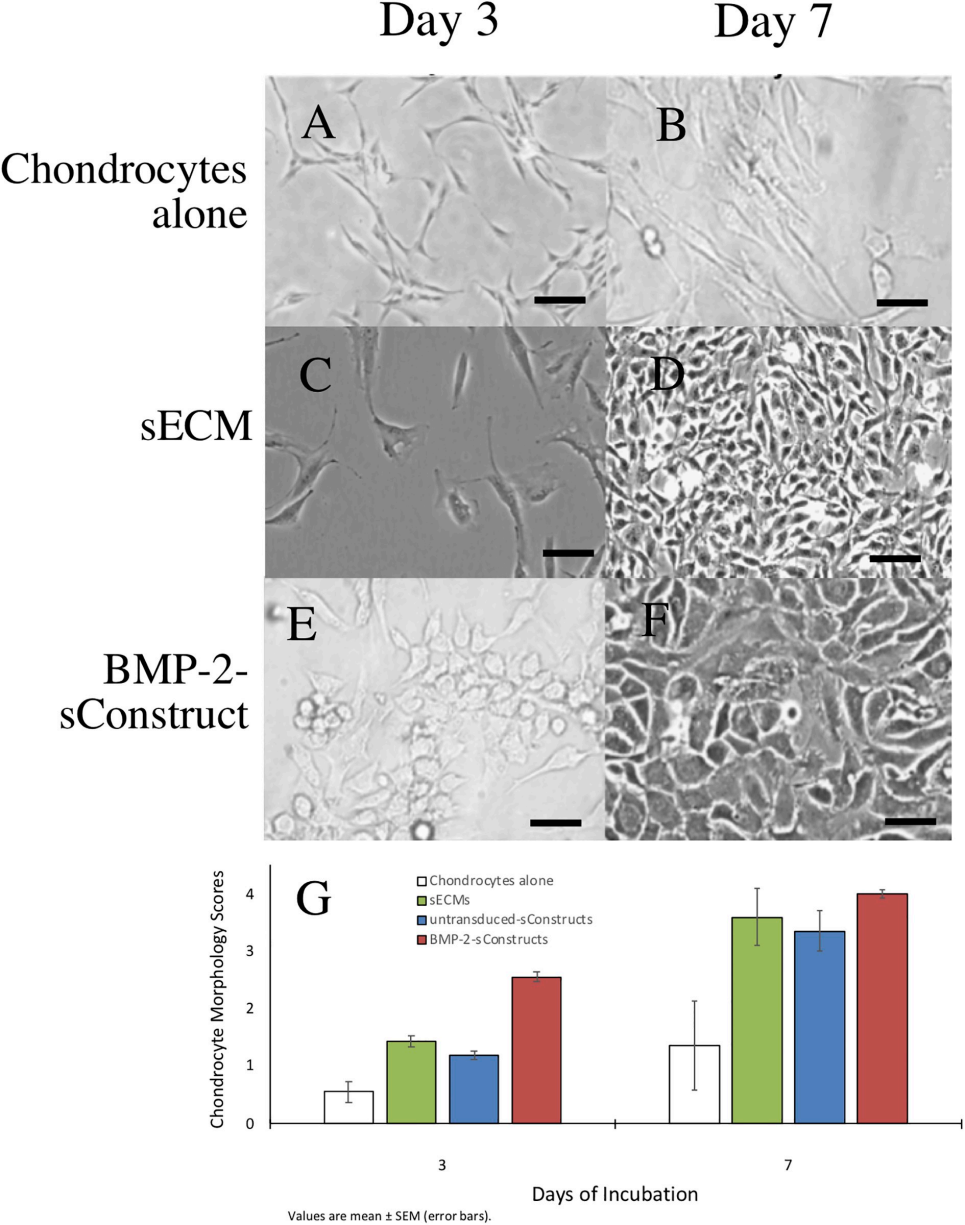
Co-cultured chondrocyte monolayers and morphology scores. Chondrocytes co-cultured with sECM or BMP-2-sConstructs. Selected photomicrographs, 20X, (A-F) and scores (G) from chondrocytes cultured alone (white), with sECM (green), untransduced-sConstructs (blue) or BMP-2-sConstructs (red) on day 3 and 7.

Chondrocytes changed from elongated shaped cells, with relatively low cytoplasm-to-nucleus ratio to a chondrocyte phenotype; larger polyhedral-shaped cells with a high cytoplasm-to-nucleus ratio (Fig 3A–3F). The chondrogenesis was most dramatic in the BMP-2-sConstructs (Fig 3E and 3F). In the BMP-2-sConstruct co-cultures, already at day 3 had significant numbers of chondrocyte phenotype cells and by day 7 all cells had changed. Chondrocyte change as measured by morphology scores (Fig 3G) followed the same pattern as chondrocyte cell counts, BMP-2-sConstructs > untransduced-sConstructs > sECMs) reflecting the influence of the bioactive biologic preparations in the insert had on the chondrocytes.

#### Chondrocyte impact on sConstructs

**Fig 4:**
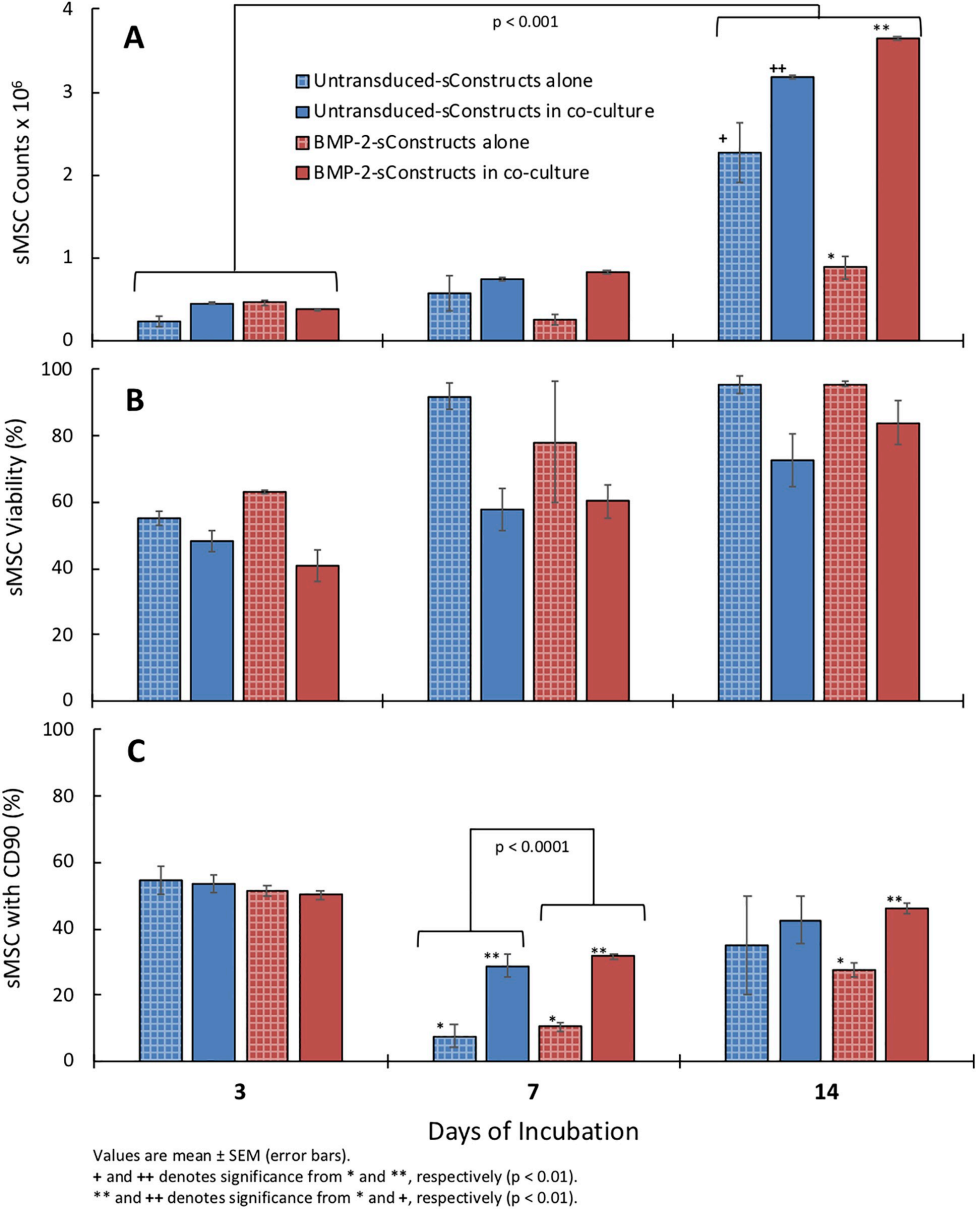
Co-culture sConstruct MSC soluble biomarker concentrations. HA (A), PG (B) and BMP-2 (C) from chondrocytes alone (white), sECM alone (green hatched), sECM chondrocyte co-culture (green), untransduced-sConstructs alone (blue hatched), untransduced-sConstructs chondrocyte co-cultures (blue) and BMP-2-sConstructs alone (red hatched), and BMP-2-sConstructs chondrocyte co-cultures (red) from media collected 1–3, 4–7, and 8–14 days.

Presence of chondrocytes in the co-cultures significantly increased the sMSC counts and % sMSC CD90 positive cells than sMSC in groups cultured without the chondrocytes. (p<0.001; Fig 4) This reflects an influence of chondrocytes on the sMSC proliferation and maturation. Specifically, untransduced-sConstructs and transduced-sConstructs (Fig 4A), rebounded by day 14 to a 2.5-fold (p < 0.001) increase in untransduced-sConstructs and 2.9-fold (p < 0.001) increase in BMP-2-sConstructs when cultured with chondrocytes, significantly greater than without chondrocytes. Cell viability was not influenced by presence or absence of chondrocytes (Fig 4B).

**Fig 5:**
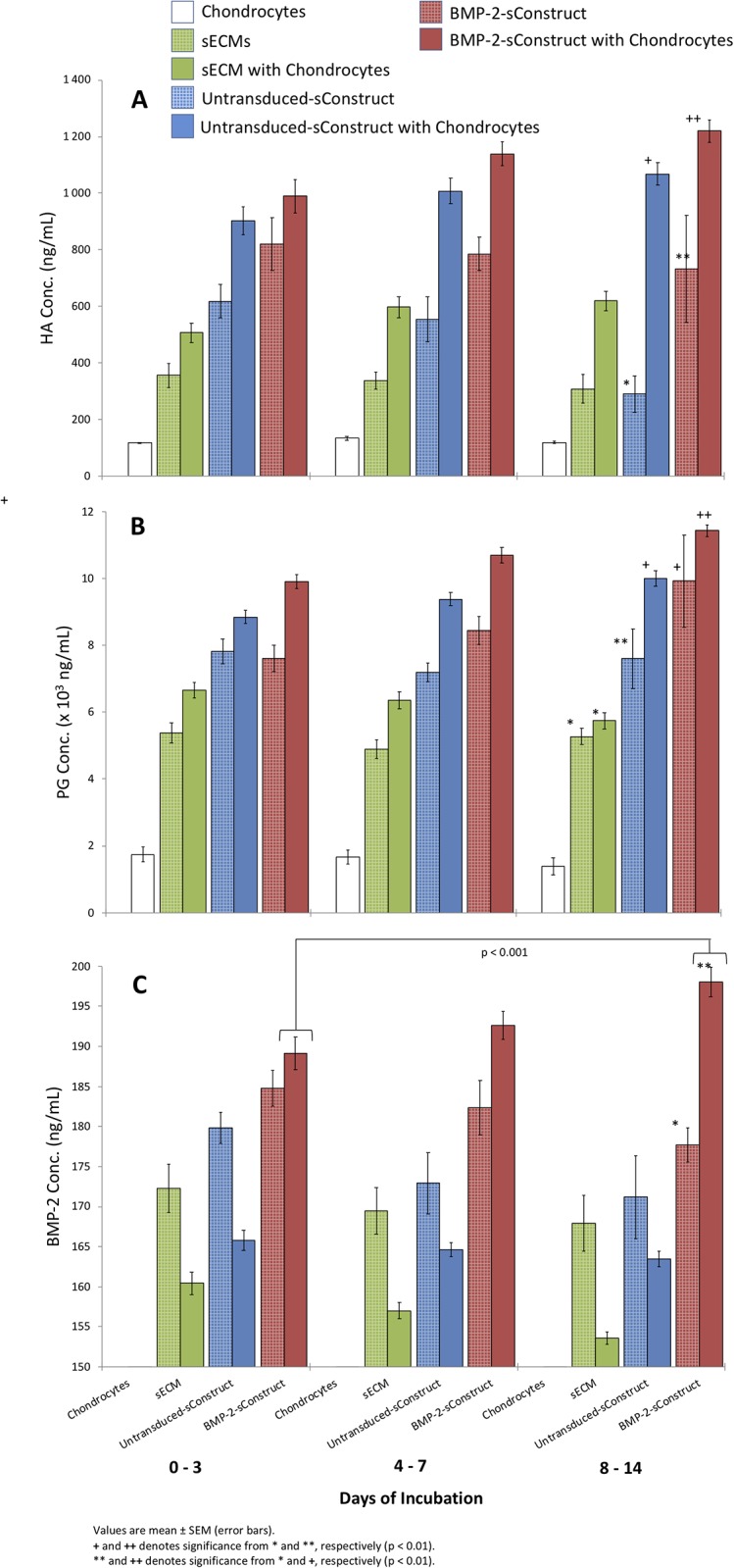
Co-culture sConstruct sMSC count, viability, and maturity. Total cell count (A), % 7AAD (B), and % CD90 expression (C) with untransduced-sConstructs alone (blue hatched), untransduced-sConstructs in co-culture (blue), BMP-2-sConstructs alone (red hatched) and BMP-2-sConstructs in co-culture (red) after incubation for 3, 7, and 14 days.

Presence of chondrocytes in the co-cultures also significantly increased the levels of soluble biomediators in media (HA, PG, BMP-2) from sECM and sConstructs compared to sECMs and sConstructs cultured without chondrocytes. (p<0.01; Fig 5) This confirmed the feedback influence that the chondrocytes also had on the biologic preparation in the insert, resulting in greater expression of synovial phenotype bioactive mediators. After 14 days, it is clear that BMP-2-sConstructs > untransduced-Constructs > sECM in production of HA (p < 0.01), PG (p < 0.05), and BMP-2 (p < 0.001).

Chondrocytes alone had a residual level of PG concentration (Fig 5B) and the significant increase in PG by the sECM and sConstructs with chondrocytes is of the same magnitude as produced by chondrocytes alone. However, for HA, the increase is much greater than the low level produced by chondrocytes alone, confirming release of HA by sECM and higher production in sConstructs, enhanced by the presence of chondrocytes. Also, noteworthy, sECMs released a low and sustained level of endogenous HA, PG, and BMP-2 during the 14 days of incubation, but significantly lower than the sConstruct-BMP-2.

In all groups, with and without chondrocyte co-culture, the sMSCs followed a growth pattern (Fig 4) that is characteristic of sMSCs infiltrating sECMs [18]. By day 3, cell counts decreased 77% for untransduced-sConstructs and 48% for transduced-sConstructs (Fig 4A), then rebounded by day 14, with both the untransduced- and transduced-sConstructs in co-culture significantly higher (p<0.01). sMSC viability as measured by 7AAD (Fig 4B) showed a less pronounced but similar pattern that returned to day 14 baseline values on Day 0 of > 80%. Cell maturity as measured by CD90 expression decreased from > 70% to < 30% in day 7, then rebounded slightly by day 14 (Fig 4C).

BMP-2-sConstruct sMSCs cell counts and cell maturity (CD90 expression) was significantly higher than untranduced-sConstructs after 14 days incubation (Fig 4A and 4C).

### In vivo studies

#### sConstruct implant impact on rat knee lesions

**Fig 6:**
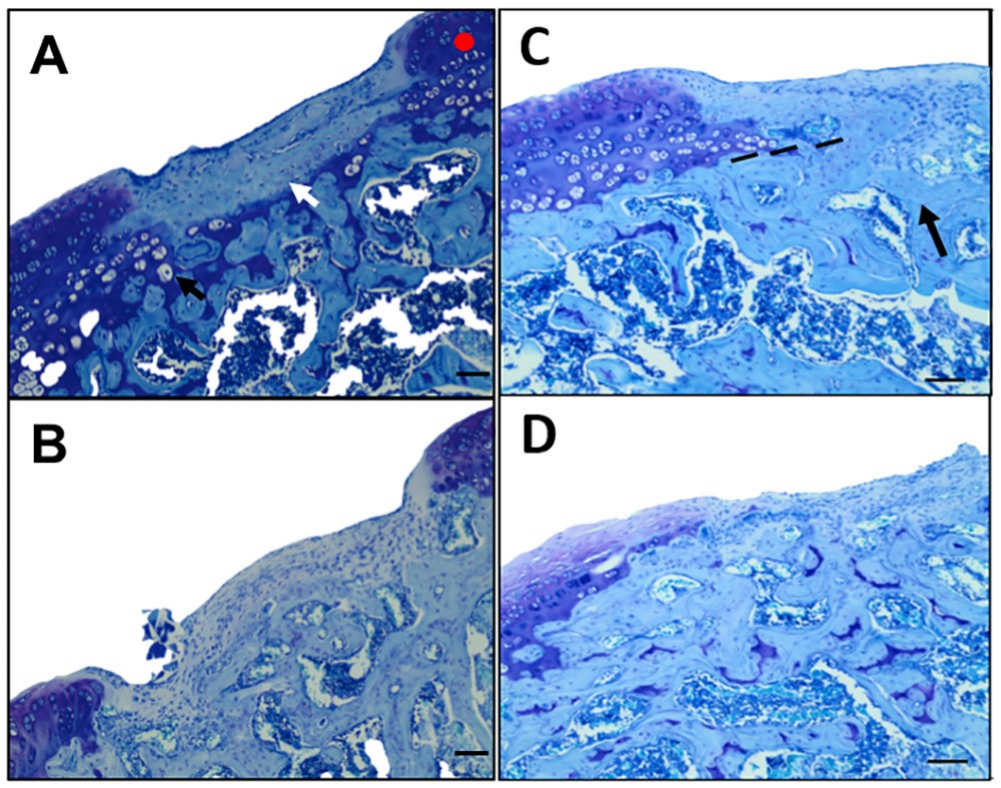
Lesion with sConstructs implants; Smooth fibrocartilage repair tissue, adjacent articular cartilage and subchondral bone repair. Illustrative (median representative) photomicrographs (40x) of histochemical toluidine blue stained sections of rat knee lesions after 5 weeks of healing. Specimens selected for the photograph had the middle histology score for that group and thereby is a mid-representative of the differences. A) Lesions with BMP-2-sConstruct implants where the white arrow (⇨) points to smooth fibrocartilage repair tissue, the black arrow (➨) to chondrocyte/lacunae duplets, and the red dot, the histochemical staining for PG. B) Image as Fig 6A but from a control knee with suture only. C) Lesions with BMP-2-sConstruct implants where black arrow (➨) points to re-established subchondral bone and dotted line (- - - - -) is re-established tidemark, D) Similar image to Fig 6C from a control knee with suture only. Scale bar: 200 μm.

Post-operative assessments were performed daily and showed no pain, chewing, inflammation, redness nor leaky wounds in the surgical area. After a 5-week healing period there were dramatic differences between lesions with sConstructs, and those with suture or sECM implants. A gross visual review of the lesion showed that both the GFP- and BMP-2-sConstruct implants produced smoother edges to the lesion, a smooth fibrocartilage that filled the lesion, less adjacent cartilage degradation and few osteophytes. Photomicrographs of lesions confirmed the gross visual review (Fig 6). Lesions with BMP-2-sConstruct implants (Fig 6A) were > 50% filled with smooth fibrocartilage repair tissue integrated with subchondral bone. Adjacent articular cartilage repair showed anabolic characteristics including chondrocyte/lacunae duplets, strong histochemical staining of PG and maintenance of cartilage thickness. Control knees (Fig 6B) with suture or sECM implants had almost no fibrocartilage filling, and an irregular subchondral bone plate without cartilage integration. The adjacent cartilage was thinner with less intense histochemical staining and there was a loss of cellularity.

In BMP-2-sConstruct implants (Fig 6C), subchondral bone was re-established, calcified chondrocytes were evident across most of the defect, the tidemark was re-establishing, and the surface of the cartilage lesion and adjacent cartilage was smoother. Control knees (Fig 6D) with only a suture had repair tissue that extended into the marrow with an irregular, less defined, tidemark area and obvious marrow changes.

**Fig 7:**
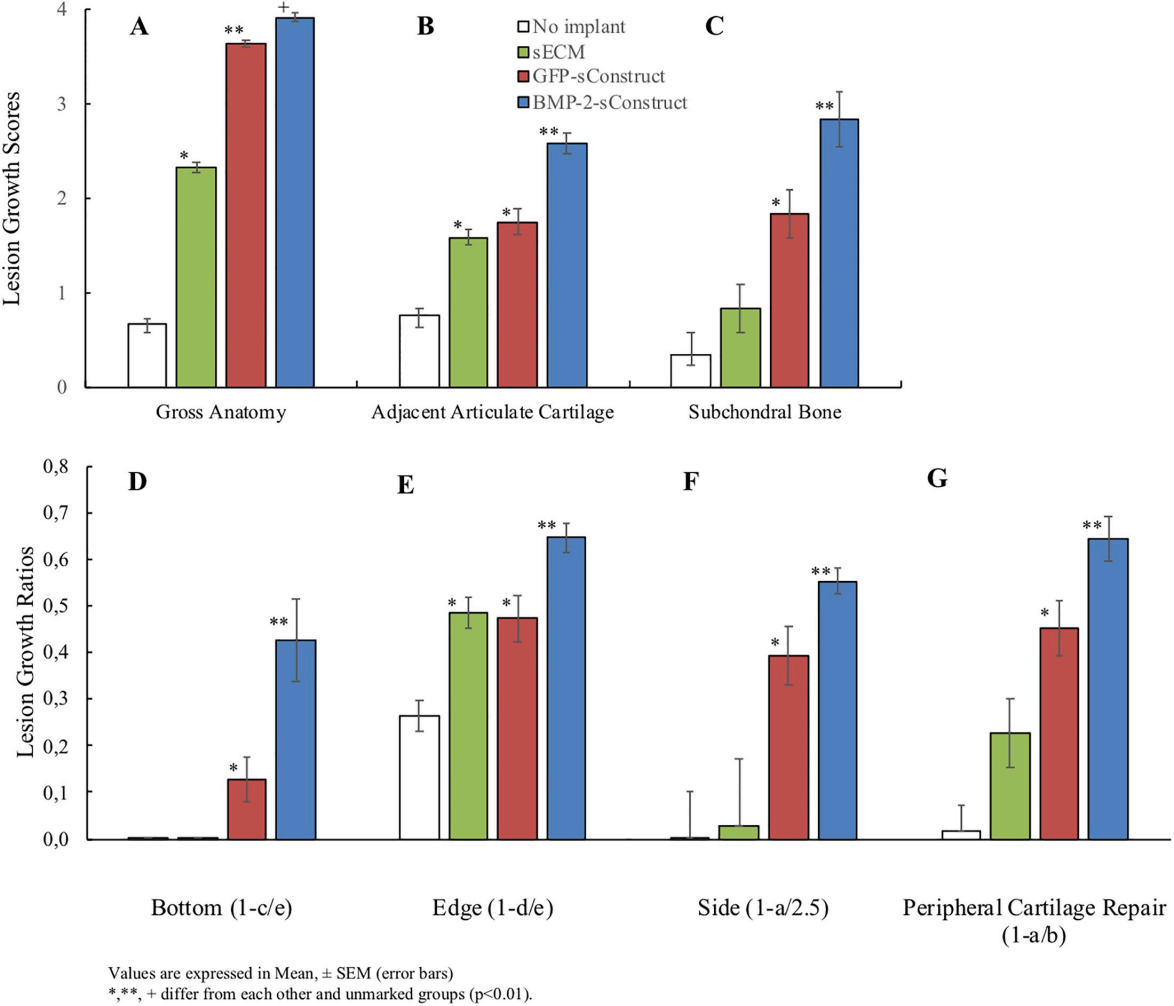
Lesion with sConstruct implants; Growth scores and filling measurements. Gross anatomy (A), articulate cartilage (B), and subchondral bone repair scores (C) and bottom (D), edge (E), sides inwards (F) and peripheral cartilage (G) measurement ratios. Lesion with no implant (white), with sECM (green), GFP-sConstructs (blue) or BMP-2-sConstructs (red).

The sConstructs demonstrated a clear pattern of repair tissue growth enhancement when the gross anatomy, adjacent articular cartilage growth and subchondral bone repair was scored; BMP-2-sConstructs > GFP-sConstructs > sECMs (Fig 7A–7C). The gross anatomy and subchondral bone repair scores (compare Fig 7A to 7C) showed a significant progression in all the samples (p < 0.01 for all implants).

To determine the amount of filling from lesion measurements (Fig 1), relative ratios were calculated and graphed (Fig 7D–7G); filling from bottom (1-c/e), edge (1-d/e), sides inwards, (1-a/2.5 mm), and peripheral cartilage repair (1-a/b). Lesion implants with BMP-2-sConstructs showed the most filling (p <0.01) followed by GFP-sConstructs. sECM implants had some enhanced growth at the edges (p <0.01) as well as in the peripheral cartilage repair (p < 0.01).

sECMs and GFP-sConstructs implants had no significant difference in the adjacent articulate cartilage scores (Fig 7B) or edge filling (Fig 7E) (p >0.05).

#### Implants in rat synovium

**Fig 8:**
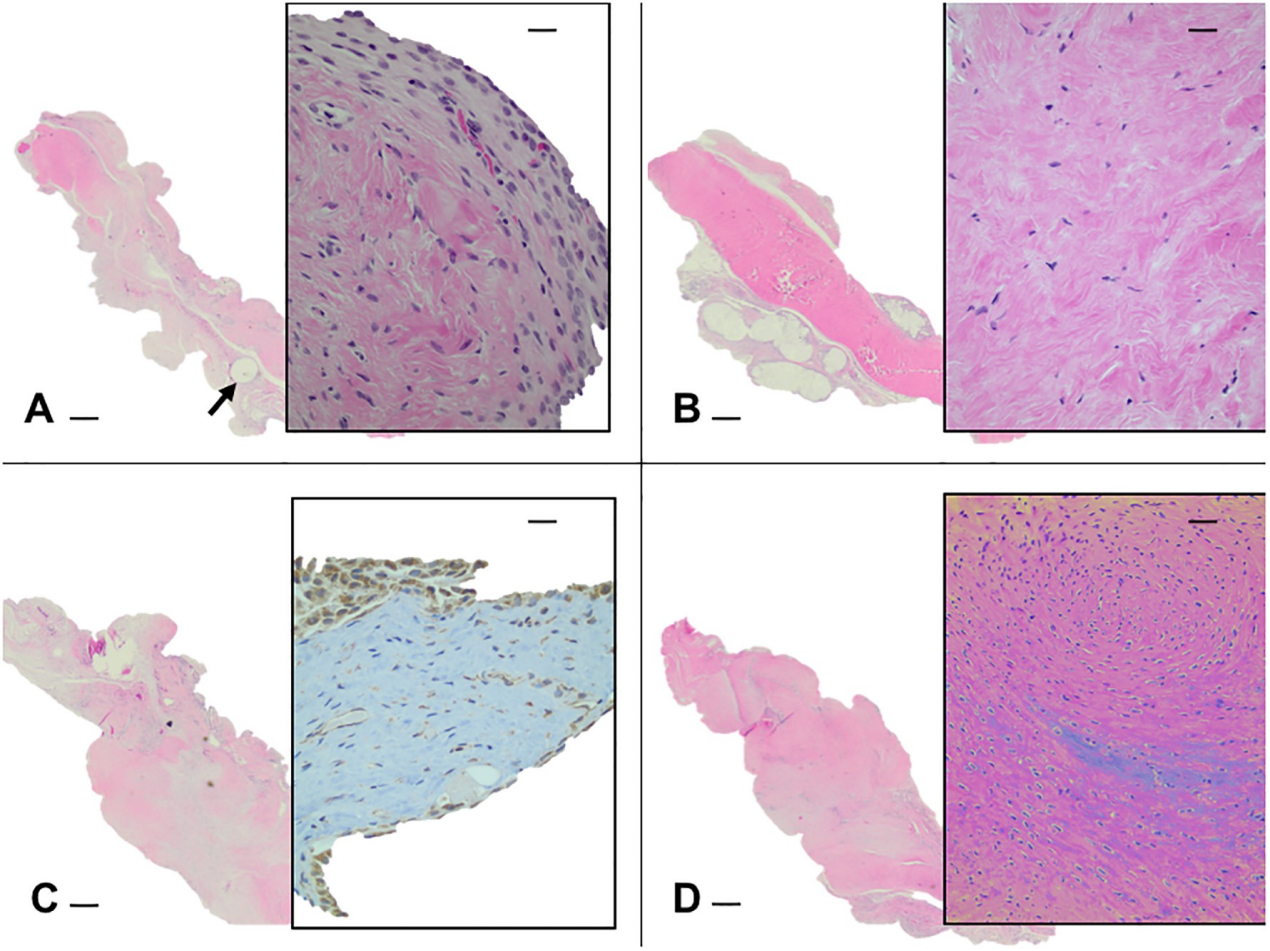
sConstructs recovered from implant site. Histophotomicrographs (2X) of implants recovered from rat knee synovium. A) suture only with suture tracks (➨), B) sECM, C) GFP-sConstruct and D) BMP-2-sConstruct. Inserts are 20X magnification and C) was stained immunohistochemically for the presence of GFP. Scale bars represent 100 μm in the main picture and 10 μm for the inserts.

Implants harvested from the rat knee synovium are shown in Fig 8. Suture only specimens had normal synovium with synovial cells layered over a loose adventia with distinctive suture tracks (Fig 8A). The sECM implantation site (Fig 8B) had a denser area of tissue, identified as the implant, with cells dispersed throughout a strongly eosinophilic scaffold. The GFP- and BMP-2-sConstruct implants (Fig 8C and 8D) showed a patch of firm tissue with less eosinophilic tissue area including focal areas of cell congregation with a significantly greater number of cells. The GFP-sConstruct implant (Fig 8C insert) had many GFP intensely stained cells (brown stained cells). When other implants were stained with GFP immunochemistry there was a mild background coloring of some cells and matrix but no intensely stained cells. BMP-2-sConstructs (Fig 8D insert) had the greatest cell density that had permeated the implant reducing collagen density.

## Discussion

This study conclusively shows that sConstructs enhance chondrogenesis *in vitro* and cartilage healing *in vivo*. It is a “proof of concept” that sMSC synovium scaffold Construct has the potential to be a vehicle creating the correct microenvironment for the repair of damaged joints to treat cartilage injury and OA.

The results strongly support a signaling loop mechanism between sConstructs and chondrocytes, both *in vitro* and *in vivo*. The sConstruct’s anabolic biofactors amplify a paracrine, restoration and maturation response of chondrocytes that, in turn, impacts the sConstruct’s own maturation and biofactor output. *In vitro*, sConstruct-chondrocyte co-cultures showed increased chondrocyte and sMSC cell counts, increased intracellular chondrocyte Collagen II and increased production of soluble HA and BMP-2, respectively. *In vivo*, rat knee lesions with sConstruct implants, showed markedly improved articular cartilage growth and subchondral bone repair. Correspondingly, resected sConstruct implants had substantial morphological changes with a marked increased cell integration.

It was striking how much the increased BMP-2 concentrations reinforced the feedback loop. BMP-2-sConstruct co-cultures boosted the chondrocyte maturation and intracellular Col II production. After 14 days of BMP-2-sConstruct co-culture, chondrocyte intracellular Col II levels were > 2-fold that of any other samples. After just 3 days of incubation, co-cultured chondrocytes were reverting to one typical of a hypertrophied cell or chondroblast. Correspondingly, BMP-2-sConstruct’s MSCs had higher cell counts and produced more HA than untransduced-sConstructs. The results also show that BMP-2 delivered an anabolic boost to sMSCs proliferation and differentiation that overcame the initial loss of viability, presumably due to cytotoxic Ad vector transduction [22] and from the seeding procedure [18]. The production of BMP-2 by BMP-2-sConstructs in co-cultures was marked, with soluble BMP-2 concentrations higher than those considered to be in the bioactive range (> 170–180 ng/mL) [38,39].

The strong effect of BMP-2 was also observed *in vivo*. BMP-2-sConstruct implants considerably improved gross anatomy, adjacent articulate cartilage growth and subchondral bone repair over that of other implants. The morphological changes of the implants were by far the most pronounced in the BMP-2-sConstruct.

The BMP-2 effects observed here are in line with previous studies. BMPs have been shown to be involved in stimulating MSC differentiation and cell recruitment, along with other functions in chondrogenesis and osteogenesis [40,41]. There was no sign of any deleterious effects of BMP-2 on cartilage [27], no bone growth or overgrowth into the synovium with implanted BMP-2-sConstructs.

sECMs seem to contain endogenous BMP-2. sECMs contained no MSCs, nor did chondrocytes alone produce BMP-2, yet significant amounts of soluble BMP-2 was found in sECMs cultured with chondrocytes or alone. It is known that ECMs can be a reservoir of biomediators [42] and it appears that the method of sECM preparation [8] does not eliminate reserves of BMP-2. BMP-2 concentrations were lower in sECM-chondrocyte co-cultures than in sECMs alone. A plausible explanation is the ability of chondrocytes to take up BMP-2 [43], thus lowering the co-culture concentration of the growth factor as compared with sECM alone.

The sECM implants, *in vivo*, caused a small but significant enhancement of lesion fill as well as a migration of endogenous cells into the sECM scaffold. This may have been caused by increased local BMP-2 concentration producing a growth environment for repair of the lesion. However, ECMs could have other endogenous growth biofactors as well as structural properties [44] that could have activated 1) changes in local chondrocytes or 2) the migration of growth inducing endogenous cells that contributed to the enhanced lesion repair.

HA acts as a boundary lubricant and an anti-inflammatory substance that inhibits the adherence of immune complexes to neutrophils and protects the synovial tissues from the attachment of inflammatory mediators [45]. sConstruct’s increased levels of HA production in co-cultures with chondrocytes, suggests they should have a positive effect on joints healing from cartilage damage. PG concentrations significantly increased for sConstructs alone [18] or as shown here in chondrocyte co-cultures. Chondrocytes alone produced PG, that could account for the observed increase in other groups.

The *in vitro* and *in vivo* results correlated remarkably well. Both showed that BMP-2-sConstruct gave the highest chondrogenesis and healing potential. Importantly, the *in vitro* and *in vivo* results had the same trend; BMP-2-sConstructs > GFP-sConstructs or untransduced-sConstructs > sECM. This study provided both *in vitro* and *in vivo* testing methods for future studies. While BMP-2 was used in this study as an example of how transduced sMSCs may impact chondrogenesis, other growth factors alone or in combination enhance chondrogenesis of sMSCs [24,46] and should be tested to improve the efficacy of sConstructs.

In this study, 1x10^6^ sMSCs were used for seeding the sConstructs in the inserts, while 1x10^3^ chondrocytes were cultured in the wells. The sMSC cell amounts were chosen because previous studies [18] had shown a cellular distribution pattern that had infiltrated the sECMs after 7 days using the 30% FBS gradient to augment the seeding process. Chondrocytes, at 1x10^3^ cells, made a confluent monolayer of morphologically transformed cells at day 7, when co-cultured with sConstructs. The surface area for cell attachment was anticipated to be greater in the complex matrix than the flat surface of the well, corresponding to the difference in seeding numbers selected. Thus, the conditions in this study gave extremely pronounced results, good for an *in vitro* test model. Other cell concentrations could be tested in the future to optimize this model.

Limitations of this study included the relatively short duration of *in vitro* incubation times. The *in vitro* 14 day was chosen because of the chondrocyte cell growth patterns. Within 7 days of co-culture, all the chondrocytes had matured, and the wells were fully covered with cells. We selected a time anticipated to show both delay or acceleration of chondrogenesis. By 14 days the sECMs were well seeded with viable cells. We also anticipated a less than two-week time period from culture to implantation at surgery, in either our rodents or in human patients in the future. We focused our efforts on this time window. Longer term studies would be needed to study toxicity or shelf-life of the sConstructs. Another limitation was the short *in vivo* incubation time. Although we selected the time point of healing of 5 weeks to catch the window to detect either a delay or acceleration of repair among our comparison groups, longer term studies would be needed to demonstration the durability of the repair cartilage and the development of OA in these knees. Current studies on OA treatments using large animal models are expected to have 6 months, or more, of observation in this final stage *in vivo* models. By restricting the treatment time, we were not able to see the overall healing effect of the sConstruct. The 5-week treatment period was also selected to allow adequate time for measurable cartilage healing, without complete degradation of the sConstruct implanted in the synovium. Further work to optimize the conditions for sConstruct implants, such as type of MSC, the seeding period prior to implantation, cell seeding number, and scaffold size all needs further study.

A limitation was the use of athymic nude rats to eliminate any contribution that might arise from an immunologic reaction by the equine sConstructs. MSCs have been recognized to be ‘immune privileged’ which is thought to enable MSC transplantation across major histocompatibility barriers and may result in the creation of “off-the-shelf” therapies consisting of MSCs grown in culture [47–49]. However, there have been some clinical trials reporting an immune response [47]. Similarly, decellularized ECM scaffolds, appropriately prepared (DNA concentration and base pair length [50]), can be implanted as xenogeneic grafts [51,52]. The combination of sECM scaffold and sMSC’s in the sConstruct have great potential as a hypoimmunogenic product that could be used allogenically. Preliminary results from our laboratory indicate there is a very minor immune response when sConstructs were co-cultured with peripheral blood mono nuclear cells (PBMCs).

To further study the immune component of syngeneic verses allogenic sConstructs, MSCs should be thoroughly tested in immunocompetent models. Understanding the mechanisms of scaffold-cell interactions could refine the treatment process by balancing anabolic and anti-inflammatory pathways. Proteomic data on the sECM will direct future work on the mechanism of implantation effects on cartilage.

In conclusion, the synergistic potential of sConstructs on distant articular cartilage has been clearly demonstrated. To evaluate modified sConstructs, this work has developed a fast *in vitro* method that correlates well with *in vivo* results. It is a “proof of concept” that sConstructs could be used as a possible treatment for OA in the future.

## Supporting information

S1 File Raw data. This file with multiple worksheets contains the raw data used to produce Figs 2, 3, 4, 5 and 7.
(XLSX)
